# Hemodynamic variables and mortality in cardiogenic shock: a retrospective cohort study

**DOI:** 10.1186/cc8114

**Published:** 2009-10-02

**Authors:** Christian Torgersen, Christian A Schmittinger, Sarah Wagner, Hanno Ulmer, Jukka Takala, Stephan M Jakob, Martin W Dünser

**Affiliations:** 1 Department of Anaesthesiology and Critical Care Medicine, Innsbruck Medical University, Anichstrasse 35, 6020 Innsbruck, Austria; 2 Department of Intensive Care Medicine, Inselspital, Medical University of Bern, Freiburgstrasse, 3010 Bern, Switzerland; 3 Department of Medical Statistics, Computer Sciences and Health Management, Innsbruck Medical University, Schöpfstrasse 41/1, 6020 Innsbruck, Austria

## Abstract

**Introduction:**

Despite the key role of hemodynamic goals, there are few data addressing the question as to which hemodynamic variables are associated with outcome or should be targeted in cardiogenic shock patients. The aim of this study was to investigate the association between hemodynamic variables and cardiogenic shock mortality.

**Methods:**

Medical records and the patient data management system of a multidisciplinary intensive care unit (ICU) were reviewed for patients admitted because of cardiogenic shock. In all patients, the hourly variable time integral of hemodynamic variables during the first 24 hours after ICU admission was calculated. If hemodynamic variables were associated with 28-day mortality, the hourly variable time integral of drops below clinically relevant threshold levels was computed. Regression models and receiver operator characteristic analyses were calculated. All statistical models were adjusted for age, admission year, mean catecholamine doses and the Simplified Acute Physiology Score II (excluding hemodynamic counts) in order to account for the influence of age, changes in therapies during the observation period, the severity of cardiovascular failure and the severity of the underlying disease on 28-day mortality.

**Results:**

One-hundred and nineteen patients were included. Cardiac index (CI) (*P *= 0.01) and cardiac power index (CPI) (*P *= 0.03) were the only hemodynamic variables separately associated with mortality. The hourly time integral of CI drops <3, 2.75 (both *P *= 0.02) and 2.5 (*P *= 0.03) L/min/m^2 ^was associated with death but not that of CI drops <2 L/min/m^2 ^or lower thresholds (all *P *> 0.05). The hourly time integral of CPI drops <0.5-0.8 W/m^2 ^(all *P *= 0.04) was associated with 28-day mortality but not that of CPI drops <0.4 W/m^2 ^or lower thresholds (all *P *> 0.05).

**Conclusions:**

During the first 24 hours after intensive care unit admission, CI and CPI are the most important hemodynamic variables separately associated with 28-day mortality in patients with cardiogenic shock. A CI of 3 L/min/m^2 ^and a CPI of 0.8 W/m^2 ^were most predictive of 28-day mortality. Since our results must be considered hypothesis-generating, randomized controlled trials are required to evaluate whether targeting these levels as early resuscitation endpoints can improve mortality in cardiogenic shock.

## Introduction

Catecholamine inotropes are the traditional pharmacologic agents used to stabilize hemodynamic function in cardiogenic shock patients [1,2]. Although catecholamines can increase systemic blood flow and ensure tissue perfusion [3], there are few beneficial effects on the heart itself. In contrast, numerous adverse effects of adrenergic agents on heart function have been reported [1,4]. These range from tachycardia/tachyarrhythmia [5] and myocardial stunning [6-8] to necrosis and apoptosis [9]. Adverse cardiac effects of catecholamines are frequently dose-dependent and may counteract re-establishment of normal heart function [1,4,7-10].

Aside from the severity of the underlying cardiac pathology, the extent of catecholamine support in cardiogenic shock patients is largely determined by the level of the prescribed hemodynamic goals. These should be set to secure tissue perfusion while minimizing adrenergic stress on the heart [1,11]. Despite the key role of hemodynamic goals, there are few data addressing the question of whether hemodynamic variables are associated with patient outcome or should be used as treatment goals in cardiogenic shock. Even less evidence exists about which endpoints of hemodynamic variables should be increased to optimize outcome. The definition of hemodynamic variables and their optimum levels for patient outcome could further help prioritize hemodynamic resuscitation, guarantee tissue perfusion and keep adrenergic stress on the healing heart as low as possible.

In this explorative, retrospective analysis, the association between hemodynamic variables and 28-day mortality as well as hemodynamic variables and indices of tissue perfusion was evaluated in 119 patients with cardiogenic shock. Additionally, we sought to identify levels of relevant hemodynamic variables to predict death at day 28. We hypothesized that one or more hemodynamic variables were associated with 28-day mortality and that certain threshold levels of these hemodynamic variables could best predict 28-day mortality.

## Materials and methods

This retrospective, explorative cohort study was performed in the 30-bed multi-disciplinary intensive care unit of the Inselspital University Hospital of Bern. Medical records from 1 March, 2005, to 30 June, 2008, were reviewed for patients admitted to the intensive care unit because of cardiogenic shock. Cardiogenic shock was defined as the simultaneous presence of all of the following criteria immediately before or during the first 24 hours after intensive care unit admission: 1) arterial hypotension (systolic arterial blood pressure below 90 mmHg or mean arterial blood pressure below 70 mmHg for 30 minutes or longer with or without therapy); 2) a cardiac index below 2 L/min/m^2 ^and a pulmonary artery occlusion pressure above 18 mmHg in patients with a pulmonary artery catheter or an acute decrease of the left ventricular ejection fraction below 40% in patients without a pulmonary artery catheter; 3) need for a continuous infusion of inotropic drugs (any dose of dobutamine, epinephrine, milrinone and/or levosimendan). Patients below the age of 18 years, patients who developed mechanical complications requiring early cardiac surgery, patients who developed cardiogenic shock after cardiac surgery, patients who required a mechanical assist device other than an intra-aortic balloon pump before or during the first 24 hours after intensive care unit admission (n = 5) and patients who developed cardiogenic shock later during the intensive care unit stay were excluded from the analysis. Presence of inclusion and absence of exclusion criteria was verified by reviewing medical charts and the patient data management system of all patients admitted to the intensive care unit with cardiogenic shock. The study protocol was approved by the Ethic Committee of the Canton of Bern, and the need for an informed consent was waived.

All study variables were extracted from the medical records and the institutional patient data management system database (Centricity Critical Care Clinisoft^®^; General Electrics, Helsinki, Finland). Routine data recording included demographic and clinical patient characteristics. Hemodynamic parameters were prospectively recorded. The system uses median filtering, which is an effective non-linear, digital filtering process to eliminate artefacts from a signal. Thus, single hemodynamic values over two minutes are summarized as a median value [12]. All laboratory results are automatically imported into the system. Drugs and fluids administered are manually entered into the database at the bedside.

### Hemodynamic therapy

Arterial, central venous and pulmonary artery catheters (Swan Ganz CCOmbo^® ^CCO/SvO_2_/VIP; Edwards Lifesciences Inc., Irvine, CA, USA) with continuous cardiac output and mixed venous oxygen saturation measurement (Vigilance^®^; Edwards Lifesciences Inc., Irvine, CA, USA) were in place in 119 (100%), 113 (95%), and 92 (77%) study patients, respectively. Arterial blood pressure measurements were preferably taken from a radial arterial line and in some patients from a femoral arterial line but never from the descending aorta through an intra-aortic balloon pump. The hemodynamic management of study patients was based on an institutional protocol, which served as a treatment guideline [13]. To maintain individual cardiac index and mixed venous oxygen saturation between 1.5 and 2.7 L/min/m^2 ^and 55 and 65%, respectively, all patients were treated with an inotropic agent. Dobutamine and epinephrine were used as first-line agents, while milrinone served as a second-line drug. During the first 24 hours after intensive care unit admission, levosimendan (no bolus injection, 0.1 to 0.2 μg/kg/min for 24 hours) was administered in four (3.4%) study patients as a last-resort therapy only. Fluid resuscitation was guided by the response of arterial blood pressure, heart rate, central venous pressure, cardiac index, mixed venous oxygen saturation, and peripheral capillary perfusion following repetitive fluid boluses. To optimize left ventricular afterload and coronary perfusion, mean arterial blood pressure was individually maintained between 50 and 75 mmHg using sodium nitroprusside to decrease or norepinephrine to increase systemic vascular resistance, as clinically indicated. If required mechanical ventilation and/or an intra-aortic balloon pump (particularly in patients with acute coronary syndrome) were used to further reduce left ventricular afterload. Packed red blood cells were transfused to increase mixed venous oxygen saturation when hemoglobin was <70 to 80 g/L.

If possible, the underlying cause of cardiogenic shock was eliminated. Patients with acute coronary syndromes were re-vascularized whenever possible using percutaneous coronary interventions. Measures were taken to keep the door-to-balloon time as short as possible and to perform coronary interventions before intensive care unit admission. Although stent implantation was prioritized, the decision to stent coronary lesions and the type of stent implanted was determined at the discretion of the operator. Before and after the procedure, patients without contraindications received a dual anti-platelet therapy (aspirin and clopidogrel) and heparin combined with abciximab in case of stent implantation.

### Demographic and clinical variables

Demographic data, premorbidities, admission year, cause of cardiogenic shock and the need for mechanical ventilation, a ventricular assist device other than an intra-aortic balloon pump (initiated >24 hours after intensive care unit admission) or renal replacement therapy during the intensive care unit stay were documented. The Simplified Acute Physiology Score (SAPS) II [14] and Sequential Organ Failure Assessment (SOFA) score [15] were calculated from worst clinical parameters during the first 24 hours after intensive care unit admission (SAPS II) and throughout the intensive care unit stay (SOFA), respectively. Length of intensive care unit and hospital stay, as well as patient outcome at intensive care unit discharge was recorded. Twenty eight day-mortality after intensive care unit admission was retrieved from institutional records, the hospital database, or in case of transfer to external institutions before day 28 by contacting these hospitals.

### Hemodynamic variables and indices of tissue perfusion

Hemodynamic variables and indices of tissue perfusion collected during the first 24 hours after intensive care unit admission were extracted from the institutional patient data management system database. Manual quality and plausibility control of individual datasets was performed to exclude artefacts (e.g. due to blood sampling via the arterial line). We have previously demonstrated that clinicians can efficiently detect artefacts in monitored trends [16]. Mean perfusion pressure (mean arterial blood pressure-central venous blood pressure) and - in patients with a pulmonary artery catheter - cardiac power index (mean arterial blood pressure × cardiac index/451) [17], coronary perfusion pressure (diastolic arterial blood pressure-pulmonary artery occlusion pressure) and systemic vascular resistance index (mean arterial blood pressure-central venous blood pressure/cardiac index × 80) were calculated.

**Figure 1:**
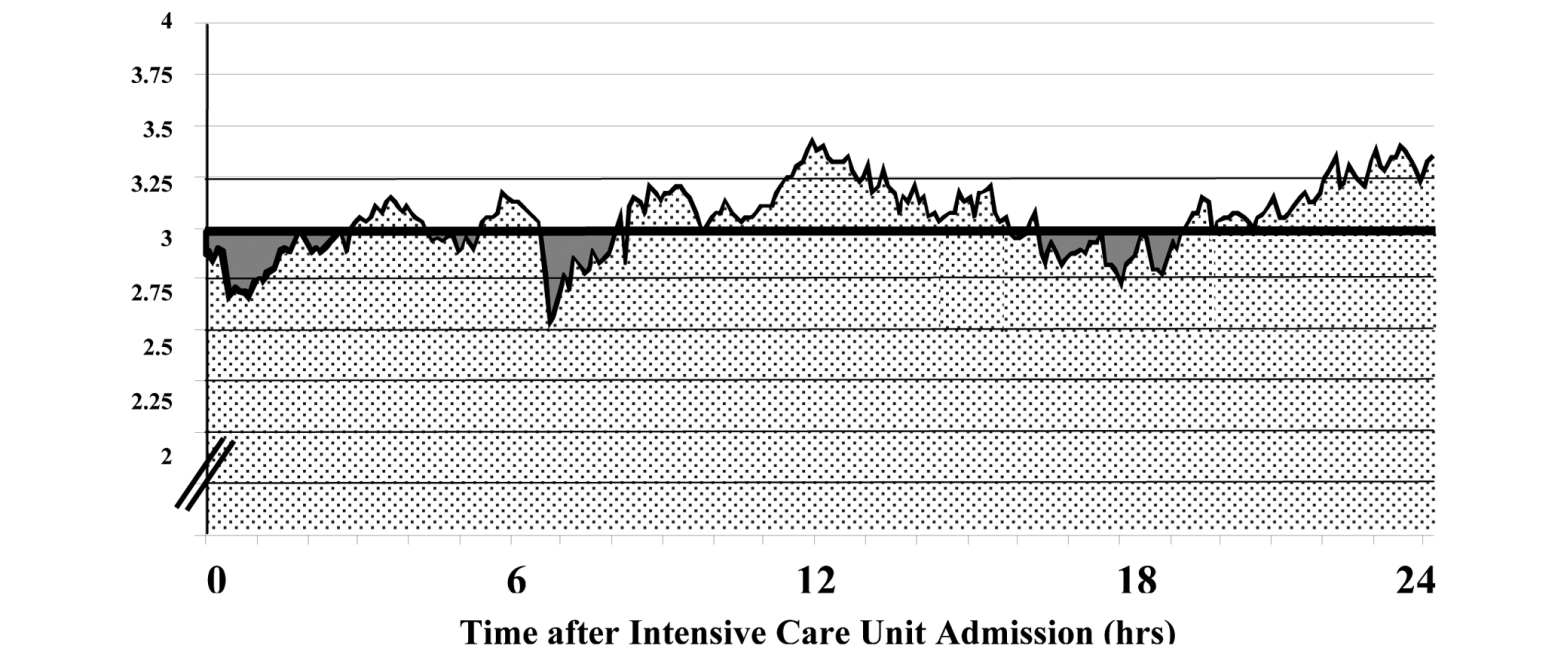
Schematic description of the cardiac index time integral and the time integral of cardiac index drops below 3 L/min/m^2 ^during the first 24 hours after intensive care unit admission.  Dotted area = cardiac index time integral. Coloured area = time integral of cardiac index drops below 3 L/min/m^2^.

Before entering the hemodynamic variables into the statistical analysis, the variable time integral during the first 24 hours was calculated for all parameters (Figure 1). Because of differences in the actual recorded time of each hemodynamic variable due to diagnostic and/or interventional procedures, the integral was normalized for the time recorded (hourly integral). In case of death during the 24 hours of observation time, hemodynamic variables during the last 30 minutes before cardiac arrest and variables recorded after the decision to withdraw life-sustaining therapy were excluded. If hemodynamic variables revealed a significant association with 28-day mortality, the hourly variable time integral of drops below clinically relevant threshold levels was calculated (Figure 1). The type and mean dose of cardiovascular drugs infused during the first 24 hours after intensive care unit admission were also documented. The most aberrant arterial lactate and base deficit levels were extracted and considered as indices of tissue perfusion.

### Study endpoints

The primary endpoint was to evaluate the association between hemodynamic variables during the first 24 hours after intensive care unit admission and 28-day mortality in cardiogenic shock. Secondary endpoints were to identify cut-off levels of those hemodynamic variables significantly associated with 28-day mortality to predict death at day 28, and to evaluate the association between hemodynamic variables and arterial lactate levels as well as base deficit as indices of tissue perfusion.

### Statistical analysis

Statistical analyses were performed using the SPSS 12.0.1. (SPSS, Chicago, IL, USA) and STATA 9.2. (StataCorp, College Station, Tx, USA) software programs. Kolmogorov Smirnov tests were applied to check for normality distribution of variables. In case of non-normal distribution, logarithmic transformation was performed. As appropriate, unpaired student's *t *and chi-squared tests were used to compare data between survivors and non-survivors.

Multivariate binary logistic regression models were calculated to evaluate the association between the hourly variable time integral of different hemodynamic variables and 28 day-mortality. Only hemodynamic variables showing no collinearity with each other (correlation coefficient <0.65) were entered into the regression models. As cardiac index and cardiac power index were strongly correlated (Pearson correlation coefficient, 0.913; *P *< 0.001) two separate multivariate logistic regression models were calculated once including cardiac index and once including cardiac power index. All models were adjusted for age, admission year, mean catecholamine (epinephrine, norepinephirne, dobutamine and milrinone) dosages and SAPS II (excluding systolic arterial blood pressure and heart rate) which were entered as linear covariates into the models in order to account for the influence of age, changes in therapies during the observation period, the severity of cardiovascular failure and the severity of the underlying disease on 28-day mortality.

To address the secondary endpoint, the area under the receiver operator characteristic (ROC) curve for the hourly variable time integral of drops below clinically relevant threshold levels of those hemodynamic variables significantly associated with 28-day mortality were determined. Additionally, sensitivity, specificity, as well as negative and positive predictive values of these variables to predict 28-day mortality was calculated from the final classification tables of the adjusted logistic regression models. The threshold level with the highest area under the ROC curve was considered to best predict 28-day mortality. Furthermore, the relative risk of death at day 28 of each threshold level was evaluated to further differentiate between the predictive value of each threshold level. To assess the association between hemodynamic variables and arterial lactate as well as base deficit, linear regression models were used. Again, these models were adjusted for age, admission year, catecholamine dosages and SAPS II (excluding systolic arterial blood pressure and heart rate). *P*-values less than 0.05 were considered to indicate statistical significance in all models. Data are given as mean values ± standard deviation, if not otherwise indicated.

## Results

**Figure 2:**
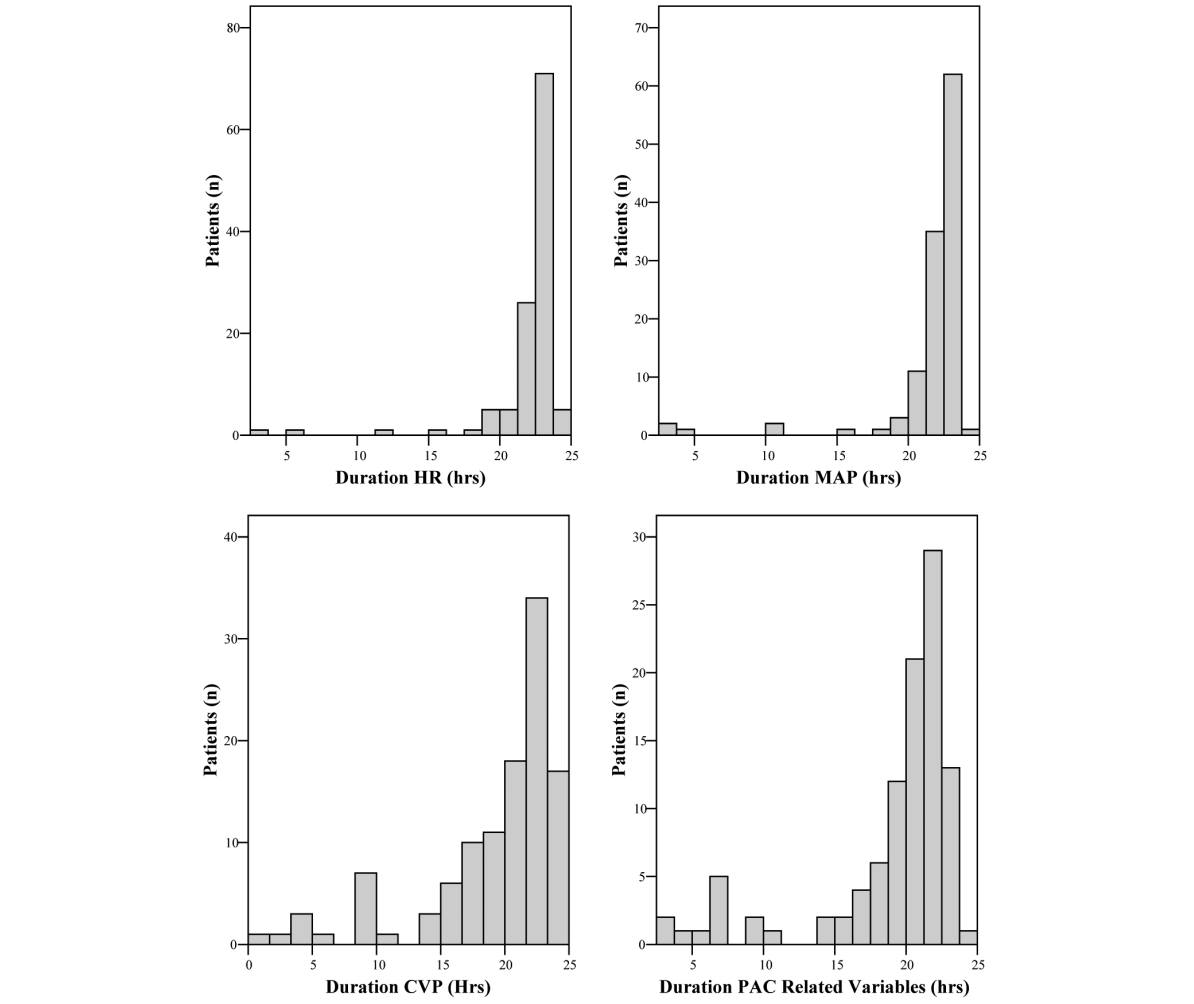
Histograms showing the time in hours of hemodynamic variable recordings in the study population.  CVP = central venous pressure; HR = heart rate; MAP = mean arterial blood pressure; PAC = pulmonary artery catheter.

During the observation period, 11,172 patients were admitted to the intensive care unit. Five patients were excluded because they received a mechanical assist device before or during the first 24 hours after intensive care unit admission. One hundred and nineteen patients fulfilled the inclusion criteria and were included into the analysis (Table 1). Heart rate, arterial blood pressure, central venous blood pressure/mean perfusion pressure as well as pulmonary artery catheter-related variables were recorded for 22.2 ± 2.9 hours, 21.7 ± 3.5 hours, 19.3 ± 5.3 hours and 19 ± 5 hours, respectively (Figure 2). Four patients died during the 24 hours of observation. Intensive care unit and 28-day mortality of the study population was 19.3% (23/119) and 29.4% (35/119). Seventy-four percent (n = 56) of patients with cardiogenic shock because of an acute coronary syndrome underwent a percutaneous coronary intervention. Stents were placed in 80.4% (n = 45) of these patients. The type and frequency of reperfusion therapies initiated before intensive care unit admission in patients with acute coronary syndrome as the cause of cardiogenic shock did not change during the observation period (2005, 72.7%; 2006, 70%; 2007, 73.9%; 2008, 90%; *P *= 0.66, chi-squared test). Cardiopulmonary resuscitation was performed in 18.5% (n = 22) of study patients before intensive care unit admission. Therapeutic hypothermia was not applied in these patients because of cardiogenic shock.

**Table 1:** Characteristics of the study population (n = 119)

Age (years)	67 ± 14
Male sex (%)	71 (59.7)
Premorbidities (%)	
Chronic arterial hypertension	41 (34.5)
Coronary heart disease	60 (50.4)
Congestive heart failure	37 (31.1)
Chronic atrial fibrillation	13 (10.9)
Chronic obstructive pulmonary disease	16 (13.4)
Chronic renal insufficiency	33 (27.7)
Chronic liver disease	22 (18.5)
Neoplasm	5 (4.2)
Obesity/metabolic syndrome	27 (22.7)
Cause of shock (%)	
Acute coronary syndrome	76 (63.9)
Decompensation of chronic cardiomyopathy	30 (25.2)
Cardiomyopathy of unknown etiology	6 (5)
Acute viral myocarditis	3 (2.5)
Acute arrhythmia	1 (0.8)
Mechanical complication	3 (2.5)
Source of admission (%)	
Emergency department	37 (31.1)
Other hospital	46 (38.7)
Other intensive care unit	23 (19.7)
Hospital ward	13 (10.9)
Sequential organ failure assessment	10.8 ± 3.1
Simplified acute physiology score II	52 ± 17
Need for mechanical ventilation (%)	98 (82.4)
Invasive mechanical ventilation	92 (77.3)
Non-invasive mechanical ventilation	6 (5)
Need for renal replacement therapy (%)	22 (18.5)
Intra-aortic balloon pump (%)	45 (37.8)
Need for ventricular assist device* (%)	14 (11.8)
Intensive care unit length of stay (days)	7.2 ± 8.7

*Ventricular assist devices include Heart Mate^®^, Tandem Heart^®^, Thoratec^® ^or Impella^® ^devices. Initiated more than 24 hours after intensive care unit admission. Data are given as mean values ± standard deviation, if not otherwise indicated.

Non-survivors at day 28 were older, had lower mean cardiac and cardiac power indices, higher epinephrine requirements, higher arterial lactate levels, SAPS II and SOFA score counts, required renal replacement therapy more often and had a shorter intensive care unit stay than survivors (Table 2). In the multivariate regression models, the hourly cardiac index and cardiac power index time integrals were the only hemodynamic variables during the first 24 hours after intensive care unit admission significantly associated with 28-day mortality (Tables 3 and 4). The hourly time integral of cardiac index and cardiac power index drops below 3 L/min/m^2 ^and 0.8 W/m^2^, respectively, revealed the highest area under the ROC curve (Table 5). The relative risk of death was positive when cardiac index and cardiac power index dropped below 3 L/min/m^2 ^and 0.8 W/m^2^, respectively. With drops below lower threshold levels, the relative risk of death at day 28 remained more or less unchanged until a cardiac index and cardiac power index of 2 L/min/m^2 ^and 0.4 W/m^2^, respectively, when a substantial increase in the relative risk of death occurred (Table 5).

**Table 2:** Demographics and clinical data of survivors and nonsurvivors at 28 days

	Survivors n = 84	Nonsurvivors n = 35	*P *value
Age (years)	65 ± 14	71 ± 12	0.01*
Male sex (%)	54 (64.3)	17 (48.6)	0.15
Heart rate (bpm)	94 ± 14	97 ± 16	0.36
SAP (mmHg)	92 ± 12	91 ± 15	0.69
MAP (mmHg)	66 ± 7	64 ± 7	0.19
DAP (mmHg)	51 ± 7	49 ± 7	0.07
MPP (mmHg)	53 ± 8	51 ± 9	0.26
CVP (mmHg)	12 ± 3	13 ± 3	0.12
MPAP† (mmHg)	28 ± 6	28 ± 6	0.89
PAOP† (mmHg)	18 ± 5	18 ± 4	0.64
CPP† (mmHg)	31 ± 7	30 ± 9	0.3
CI† (l/min/m^2^)	2.7 ± 0.5	2.4 ± 0.4	0.003*
CPI† (W/m^2^)	0.39 ± 0.08	0.34 ± 0.38	0.005*
SvO_2_† (%)	64 ± 6	62 ± 9	0.14
SVRI (dyne*s/cm^5^/m^2^)	1760 ± 664	1779 ± 357	0.89
Epinephrine# n = 50 (μg/h)	61 ± 130	195 ± 317	0.03*
Norepinephrine# n = 37 (μg/h)	26 ± 85	17 ± 54	0.6
Dobutamine# n = 89 (mg/h)	8 ± 7	10 ± 8	0.11
Milrinone# n = 14 (mg/h)	0.07 ± 0.24	0.07 ± 0.25	0.99
Nitroprusside# (mg/h)	1.87 ± 3.68	1.49 ± 2.75	0.6
Arterial lactate§ (mmol/l)	4.1 ± 3.3	6.4 ± 4	0.002*
Troponin T§ (μg/l)	54 ± 103	114 ± 228	0.19
RRT (%)	10 (11.9)	12 (34.3)	0.008*
SOFA score§	10 ± 3	12 ± 3	0.007*
SAPS II	49 ± 15	61 ± 17	< 0.001*
ICU LOS (days)	8.1 ± 9.8	5 ± 4.6	0.02*

Hemodynamic parameters reflect mean values during the first 24 hours after ICU admission.

* significant difference between survivors and nonsurvivors; † 92 (77.2%) patients were monitored with a pulmonary arterial catheter; # mean hourly dosage during the first 24 hours after ICU admission; § maximum values during the ICU stay.

Data are given as mean values ± standard deviation, if not otherwise indicated.

CI = cardiac index; CPI = cardiac power index; CPP = coronary perfusion pressure; CVP = central venous blood pressure; DAP = diastolic arterial blood pressure; ICU = intensive care unit; LOS = length of stay; MAP = mean arterial blood pressure; MPAP = mean pulmonary arterial blood pressure; MPP = mean perfusion pressure; PAOP = pulmonary arterial occlusion pressure; RRT = need for renal replacement therapy; SAP = systolic arterial blood pressure; SOFA = sequential organ failure assessment; SvO_2 _= mixed venous oxygen saturation; SVRI = systemic vascular resistance index.

**Table 3:** Separate adjusted logistic regression models to detect associations between single hemodynamic variables and 28-day mortality

	Wald	RR	95% Con Int	*P *value
CI time integral† (l/m^2^/h)	6.097	0.972	0.951-0.994	0.01*
CPI time integral† (W/m^2 ^*min/h)	4.491	0.864	0.755-0.989	0.03*
SvO2 time integral† (%*min/h)	2.315	0.999	0.998-1	0.13
SVRI time integral† (dyne*s/cm5/m^2 ^*min/h)	1.776	1	1-1	0.18
MPP time integral (mmHg*min/h)	0.999	1.001	0.999-1.002	0.32
HR time integral (bmp*min/h)	0.972	1	1-1.001	0.32
MAP time integral (mmHg*min/h)	0.178	1	0.999-1.002	0.67
MPAP time integral† (mmHg*min/h)	0.158	1	0.998-1.001	0.69
SAPS time integral (mmHg*min/h)	0.107	1	1-1.001	0.46
CVP time integral (mmHg*min/h)	0.013	1	0.997-1.003	0.91
DAP time integral (mmHg*min/h)	0	1	0.999-1.001	1

Single logistic regression models were calculated for each hemodynamic variable and were each adjusted for age, admission year, mean catecholamine (epinephrine, norepinephrine, dobutamine and milrinone) dosages and SAPS II (excl. the systolic arterial blood pressure and heart rate count). Variables are ranked (top to bottom) according to the value of the Wald statistics. * significant association with 28 day-mortality; † 92 (77.2%) patients were monitored with a pulmonary arterial catheter.

CI = cardiac index; Con Int = confidence interval; CPI = cardiac power index; CVP = central venous blood pressure; HP = hourly portion; MAP = mean arterial blood pressure; MPAP = mean pulmonary arterial blood pressure; RR = relative risk; SAPS II = Simplified Acute Physiology Score II (excl. heart rate and systolic arterial blood pressure counts); SvO2 = mixed venous oxygen saturation; SVRI = systemic vascular resistance index.

**Table 4:** Adjusted multivariate logistic regression models to detect independent associations between hemodynamic variables and 28-day mortality

	Wald	RR	95% Con Int	*P *value
	**Model I - including cardiac index**			
	
CI time integral† (l/m^2^/h)	6.658	0.914	0.854-0.979	0.01*
CVP time integral (mmHg*min/h)	4.010	0.995	0.99-1	0.06
SVRI time integral† (dyne*s/cm^5^/m^2 ^*min/h)	3.832	1	1-1	0.06
MAP time integral (mmHg*min/h)	2.914	1.003	1-1.006	0.09
HR time integral (bpm*min/h)	1.833	1.001	1-1.001	0.18
SvO2 time integral† (%*min/h)	0.069	1	0.998-1.002	0.79
MPAP time integral† (mmHg*min/h)	0.001	1	0.998-1.002	0.97
				
	
	**Model II - including cardiac power index**			
	
CPI time integral† (W/m^2 ^*min/h)	6.281	0.648	0.462-0.91	0.01*
MAP time integral (mmHg*min/h)	4.109	1.003	1-1.007	0.06
CVP time integral (mmHg*min/h)	3.076	0.996	0.991-1	0.08
SVRI time integral† (dyne*s/cm^5^/m^2 ^*min/h)	2.739	1	1-1	0.1
HR time integral (bpm*min/h)	2.072	1.001	1-1.001	0.15
MPAP time integral† (mmHg*min/h)	0.086	1	0.998-1.002	0.77
SVO2 time integral† (%*min/h)	0.002	1	0.998-1.002	0.97

All models were adjusted for age, admission year, mean catecholamine (epinephrine, norepinephrine, dobutamine, and milrinone) dosages and SAPS II (excl. the systolic arterial blood pressure and heart rate count). Variables are ranked (top to bottom) according to the value of the Wald statistics. * significant association with 28 day-mortality; † 92 (77.2%) patients were monitored with a pulmonary arterial catheter.

CI = cardiac index; Con Int = confidence interval; CPI = cardiac power index; CVP = central venous blood pressure; HP = hourly portion; HR = heart rate; MAP = mean arterial blood pressure; MPAP = mean pulmonary arterial blood pressure; RR = relative risk; SvO2 = mixed venous oxygen saturation; SVRI = systemic vascular resistance index.

**Table 5:** Association between different cardiac index/cardiac power index levels and 28-day mortality

	n (%)	AUC ROC	Sens (%)	Spec (%)	PPV (%)	NPV (%)	RR	95% Con Int
HP between different CI levels								
HTI of CI drops <3.75 l/min/m^2^	92 (100)	0.80	42.9	90.5	66.7	78.1	0.98	0.96-1.00
HTI of CI drops <3.50 l/min/m^2^	92 (100)	0.80	42.9	90.5	66.7	78.1	0.98	0.96-1.00
HTI of CI drops <3.25 l/min/m^2^	92 (100)	0.80	42.9	90.5	66.7	78.1	0.98	0.96-1.00
HTI of CI drops <3.00 l/min/m^2^	92 (100)	0.81	46.4	92.1	72.2	79.5	1.04	1.01-1.07
HTI of CI drops <2.75 l/min/m^2^_	87 (94.6)	0.80	46.4	92.1	72.2	79.5	1.04	1.01-1.08
HTI of CI drops <2.50 l/min/m^2^	84 (91.3)	0.79	39.3	92.1	68.6	77.3	1.05	1.00-1.10
HTI of CI drops <2.25 l/min/m^2^	73 (79.3)	0.78	39.3	92.1	68.8	77.3	1.06	0.99-1.14
HTI of CI drops <2.00 l/min/m^2^	58 (63)	0.77	35.7	90.5	62.5	76.0	1.11	0.96-1.27
HTI of CI drops <1.75 l/min/m^2^	38 (41.3)	0.77	39.3	93.7	73.3	77.6	1.34	0.95-1.89
HTI of CI drops <1.50 l/min/m^2^	22 (23.9)	0.76	32.1	92.1	64.3	75.3	1.46	0.69-3.10
HP below different CPI levels								
HTI of CPI drops <1.2 W/m^2^	92 (100)	0.81	39.3	88.9	61.1	76.7	0.86	0.75-0.98
HTI of CPI drops <1.1 W/m^2^	92 (100)	0.81	39.3	88.9	61.1	76.7	0.86	0.75-0.98
HTI of CPI drops <1.0 W/m^2^	92 (100)	0.81	39.3	88.9	61.1	76.7	0.86	0.75-0.98
HTI of CPI drops <0.9 W/m^2^	92 (100)	0.81	39.3	88.9	61.1	76.7	0.86	0.75-0.98
HTI of CPI drops <0.8 W/m^2^	92 (100)	0.81	39.3	88.9	61.1	76.7	1.16	1.01-1.33
HTI of CPI drops <0.7 W/m^2^	92 (100)	0.80	39.3	88.9	61.1	76.7	1.16	1.01-1.33
HTI of CPI drops <0.6 W/m^2^	91 (98.9)	0.80	39.3	88.9	61.1	76.7	1.16	1.01-1.33
HTI of CPI drops <0.5 W/m^2^	91 (98.9)	0.79	39.3	90.5	64.7	77.0	1.17	1.00-1.35
HTI of CPI drops <0.4 W/m^2^	91 (98.9)	0.79	39.3	90.5	64.7	77.0	1.20	0.98-1.47
HTI of CPI drops <0.3 W/m^2^	69 (75)	0.78	35.7	92.1	66.7	76.3	1.46	0.86-2.47

Single Receiver Operating Characteristic Curve Models were based on logistic regression models adjusted for age, admission year, mean catecholamine (epinephrine, norepinephrine, dobutamine, and milrinone) dosages and disease severity as assessed by the Simplified Acute Physiology Score II (excl. heart rate and systolic arterial blood pressure counts).

AUC ROC = area under the receiver operating characteristic curve; CI = cardiac index; Con Int = confidence interval; CPI = cardiac power index; HTI = hourly time integral; NPV = negative predictive value; PPV = positive predictive value; RR = relative risk; Sens = sensitivity; Spec = specificity.

Of all hemodynamic variables during the first 24 hours after intensive care unit admission, only the hourly cardiac index time integral was associated with base deficit (standardized Beta coefficient, 0.176; *P *= 0.04). No hemodynamic variable was associated with arterial lactate levels but epinephrine (standardized Beta coefficient, 0.341; *P *= 0.002) and norepinephrine doses were associated (standardized Beta coefficient, 0.517; *P *< 0.001).

## Discussion

In this retrospective analysis, cardiac index and cardiac power index were separately associated with 28-day mortality in 119 cardiogenic shock patients. A cardiac index of 3 L/min/m^2 ^and a cardiac power index of 0.8 W/m^2 ^during the first 24 hours after intensive care unit admission were best predictive of 28-day mortality. Cardiac index was associated with base deficit. Despite the fact that almost two-thirds of the study population developed cardiogenic shock as a result of an acute coronary syndrome, 28-day mortality was comparatively low [18-22]. This could be attributable to early and aggressive interventional measures to re-vascularize ischemic myocardium.

As therapeutic interventions during the early phase of cardiogenic shock are crucial for survival [18,19], we chose to investigate the association between hemodynamic variables during the first 24 hours after intensive care unit admission and outcome. However, it must be considered that the first 24 hours of intensive care unit therapy usually do not represent the first 24 hours of the disease process. This led to a certain lead-time bias in our analysis which is difficult to quantify and may have influenced the association between hemodynamic variables and mortality. Similarly, our analysis does not take the influence of hemodynamic changes occurring more than 24 hours after intensive care unit admission on mortality into account. On the other hand, a major strength of our analysis is that it assessed variable time integrals instead of single or averaged absolute values of different hemodynamic parameters as so far evaluated in previous clinical studies [20-22]. This variable integrates the influence of two important dimensions, namely the duration and extent of hemodynamic changes, on indices of tissue perfusion and mortality.

Of all the hemodynamic variables, cardiac index and cardiac power index were significantly associated with 28-day mortality in our cardiogenic shock population. As reflected by the association between cardiac index and base deficit, it appears that this association is at least partly related to tissue perfusion. These observations are in accordance with previous studies [20-22] and the current pathophysiologic understanding of cardiogenic shock [11]. Similar to our results, Fincke and colleagues analysed 541 cardiogenic shock patients of the SHOCK trial registry and observed that cardiac power was the strongest independent correlate of in-hospital mortality [20]. Another *post hoc *analysis of a large acute myocardial infarction database reported that cardiac output, pulmonary artery occlusion pressure and mean arterial blood pressure were associated with 30-day mortality in cardiogenic shock [21]. Other authors found similar results [22]. In contrast to these studies, which analysed hemodynamic variables measured at arbitrarily selected time points, our analysis evaluated continuous measurements during the first 24 hours after intensive care unit admission and thereby allowed the investigation of the association between the evolution of hemodynamic variables over time and outcome in cardiogenic shock.

Furthermore, statistical models applied in this analysis were all adjusted for age, admission year, catecholamine dosages and SAPS II to account for the influence of age, changes in therapies during the observation period, the severity of cardiovascular failure and the severity of the underlying disease on 28-day mortality. Therefore, our results may better reflect the true impact of hemodynamic variables on indices of tissue perfusion and mortality than earlier studies [18-20]. Nonetheless we cannot exclude that other variables not included in the regression models influenced the association between hemodynamic variables and mortality. Additionally, it must be considered that although our models were adjusted for catecholamine requirements, cardiac index or cardiac power index may not be fully comparable between study patients receiving low- or high-dosed catecholamine infusions.

Although the association between cardiac index, cardiac power index and mortality in cardiogenic shock may be expected, none of the hemodynamic variables commonly measured was associated with outcome in our analysis. It is conceivable that some variables (e.g. mean arterial blood pressure, central venous blood pressure or systemic vascular resistance index) may have been significant had more patients been included. Moreover, these variables were used as endpoints of resuscitation and could underlie a certain treatment bias. Given the pathophysiology of cardiogenic shock, cardiac index and cardiac power index could partly reflect the failure of hemodynamic interventions to influence these hemodynamic endpoints. Although only statistically non-collinear hemodynamic variables were entered into the multivariate regression model, it is also likely that a clinical correlation exists between most hemodynamic variables. Therefore, collinearity may be an inherent problem of multivariate analyses including different hemodynamic variables. However, supporting the main results of our analysis, cardiac index and cardiac power index were significant and showed the strongest association with 28-day mortality in both regression models.

According to the Wald statistics of the regression models, a certain priority rank order for the early resuscitation of cardiogenic shock patients could be established. Based on this, it appears that early hemodynamic resuscitation should focus on increasing systemic blood flow during cardiogenic shock. Furthermore, it may be hypothesized that rising systemic vascular resistance simply to maintain arterial blood pressure may not be beneficial. Accordingly, only an early increase of systemic blood flow was associated with survival in this study population.

Considering the double-edged effects of catecholamines on the heart and tissue perfusion [1,4-11], it is a central clinical question of to what levels systemic blood flow should be increased to improve mortality. As suggested by the comparison between survivors and non-survivors in our analysis as well as by results of previous studies [23], infusion of epinephrine may be particularly harmful. In view of the fact that this study was retrospective and explorative, our results must be considered as hypothesis generating. Accordingly, the adjusted models suggest that a cardiac index of 3 L/min/m^2 ^and a cardiac power index of 0.8 W/m^2 ^were best predictive of 28-day mortality in our study population. Considering that the relative risk of death at day 28 turned positive when cardiac index and cardiac power index dropped below 3 L/min/m^2 ^and 0.8 W/m^2^, respectively, and substantially increased with cardiac index drops below 2 L/min/m^2 ^and cardiac power index drops below 0.4 W/m^2^, it is likely that a clinically relevant threshold level for 28-day mortality exists between a cardiac index of 2-3 L/min/m^2 ^and a cardiac power index between 0.8 and 0.4 W/m^2^. However, considering the reduced number of patients experiencing cardiac index and cardiac power index drops below very low threshold levels, these results must be interpreted with caution and need to be confirmed in a larger patient population. Comparable cut-off values for cardiac output (5.1 L/min ~ about 2.9 L/min/m^2 ^in an adult with 1.73 m^2 ^body surface area) and cardiac power output (1 W ~ about 0.58 W/m^2 ^in an adult with 1.73 m^2 ^BSA) were reported [21,22]. However, these models were neither adjusted for confounding factors nor disease severity. Furthermore, it is important to note that the threshold levels suggested in our study did not represent treatment goals but were retrospectively defined. Their use as resuscitation goals in early cardiogenic shock must be evaluated in future randomized controlled trials. In such a trial, the safety of targeting these endpoints must also be evaluated. This is particularly relevant in face of the lacking positive or even negative results of previous large studies on the outcome effects of targeting supra-normal oxygen delivery in critically ill patients [24,25].

When interpreting our study results important limitations need to be considered. First, our analysis was retrospective and shortcomings such as missing values cannot be excluded despite all hemodynamic variables being prospectively recorded. Second, although inclusion criteria were present in all study patients, we cannot exclude that more of the 11,172 patients admitted to our intensive care unit during the observation period may have been considered as having cardiogenic shock by other definitions. Together with the fact that five patients who received a mechanical assist device before or during the first 24 hours after intensive care unit admission were excluded, this may represent a selection bias of our analysis. Third, although artefacts in monitored trends of hemodynamic variables were eliminated, we cannot rule out that malposition of the reference level of invasively measured blood pressures or a low signal quality index of mixed venous oxygen saturation measurements was present in some patients for a limited time. As close monitoring of the correct reference position and signal quality index is a standard operational procedure at our intensive care unit, we do not believe that this potential limitation is the reason why no significant association between certain hemodynamic variables and mortality could be identified. Fourth, measurement of base deficit and arterial lactate levels may have been insufficient to reliably evaluate global tissue perfusion. Particularly arterial lactate levels are influenced by other factors than tissue hypoxia alone [26]. As confirmed by our results, catecholamines are well known to increase arterial lactate levels either by exaggerated simulation of aerobic glycolysis and lactate production [27] or induction of tissue hypoperfusion by inappropriate vasoconstriction [28,29].

## Conclusions

During the first 24 hours after intensive care unit admission, cardiac index and cardiac power index are the most important hemodynamic variables separately associated with 28-day mortality in patients with cardiogenic shock. A cardiac index of 3 L/min/m^2 ^and a cardiac power index of 0.8 W/m^2 ^were best predictive of 28-day mortality. As our results must be considered hypothesis generating, randomized controlled trials are required to evaluate whether targeting these levels as early resuscitation endpoints can improve mortality in cardiogenic shock.

## Key messages

• Despite the key role of hemodynamic goals, there are few data addressing the question of whether hemodynamic variables are associated with patient mortality or should be used as treatment goals in cardiogenic shock.

• During the first 24 hours after intensive care unit admission, cardiac index and cardiac power index are the most important hemodynamic variables separately associated with 28-day mortality in cardiogenic shock patients.

• A cardiac index of 3 L/min/m^2 ^and a cardiac power index of 0.8 W/m^2 ^were best predictive of 28-day mortality.

• Randomized controlled trials are required to evaluate whether targeting these levels as early resuscitation endpoints can improve mortality in cardiogenic shock.

## Abbreviations

ROC: receiver operator characteristic; SAPS: Simplified Acute Physiology Score; SOFA: Sequential Organ Failure Assessment.

## Competing interests

The authors declare that they have no competing interests.

## Authors' contributions

CT designed the study, collected data, interpreted results, drafted the manuscript and revised it for important intellectual content. CAS collected data, interpreted results and revised the manuscript for important intellectual content. SW collected data, interpreted results and revised the manuscript for important intellectual content. HU analysed the data, interpreted the results and revised the manuscript for important intellectual content. JT designed the study, interpreted results and revised the manuscript for important intellectual content. SMJ designed the study, interpreted results and revised the manuscript for important intellectual content. MWD designed the study, analysed the data, interpreted results, drafted the manuscript and revised it for important intellectual content.
